# Nutritional adaptation to circadian misalignment: implications for musculoskeletal health in modern lifestyles

**DOI:** 10.3389/fnut.2026.1833858

**Published:** 2026-06-22

**Authors:** Houchen Liu, Yuxuan Xin

**Affiliations:** 1 Department of Traumatology, Siegfried Weller Institute, BG-Klinik Tübingen, Eberhard Karls University, Tübingen, Germany; 2 Department of Orthopedic Surgery, The First Affiliated Hospital of Shandong First Medical University & Shandong Provincial Qianfoshan Hospital, Jinan, China

**Keywords:** bone metabolism, chrononutrition, circadian disruption, circadian rhythm, musculoskeletal health, skeletal muscle

## Abstract

Circadian rhythms regulate metabolic, endocrine, and cellular processes essential for maintaining musculoskeletal health. Accumulating experimental and epidemiological evidence indicates that circadian disruption, driven by modern lifestyle factors such as shift work, sleep restriction, and irregular eating patterns, is associated with an increased risk of musculoskeletal disorders. This narrative review summarizes current evidence linking circadian misalignment to alterations in bone, skeletal muscle, and cartilage biology. At the mechanistic level, disruption of core clock gene expression and desynchronization between central and peripheral clocks lead to hormonal imbalance, metabolic dysfunction, and chronic low-grade inflammation. These pathways are associated with bone loss, impaired muscle protein synthesis, and accelerated cartilage degeneration. Findings from both animal and human studies support associations with osteoporosis, sarcopenia, and osteoarthritis. Chrononutrition has emerged as a potential strategy to mitigate these effects. Approaches such as time-restricted eating, alignment of food intake with the biological day, and optimized protein distribution may help restore circadian alignment and improve musculoskeletal outcomes. In conclusion, integrating circadian biology with nutritional timing provides a promising framework for the prevention and management of musculoskeletal disorders, although large-scale human interventional trials with validated clinical endpoints are needed to confirm benefit.

## 1 Introduction

Musculoskeletal disorders—including osteoporosis, sarcopenia, osteoarthritis, and chronic musculoskeletal pain—are among the leading causes of disability worldwide and represent a major public health challenge. According to the Global Burden of Disease study, musculoskeletal conditions affect more than 1.71 billion people globally and account for approximately 21% of years lived with disability (1). These conditions impose considerable economic burdens on healthcare systems (2, 3). Traditionally, research has focused on disease-specific mechanisms such as hormonal dysregulation, inflammatory processes, mechanical loading, and age-related degeneration (4, 5). However, accumulating evidence indicates that lifestyle-related factors—including sleep patterns, dietary behavior, and circadian rhythms—also play critical roles in maintaining musculoskeletal health (6, 7).

The circadian system is an evolutionarily conserved biological timing network that synchronizes physiological processes with the approximately 24-h environmental light–dark cycle (8). In mammals, circadian rhythms are coordinated by a hierarchical system comprising a central pacemaker in the suprachiasmatic nucleus (SCN) and peripheral clocks present in nearly all tissues. These rhythms arise from transcription–translation feedback loops involving core clock genes such as CLOCK, BMAL1, PER, and CRY (9, 10). Through these mechanisms, circadian clocks regulate energy metabolism, endocrine secretion, immune responses, and cellular repair processes. Bone remodeling, skeletal muscle protein turnover, cartilage matrix maintenance, and inflammatory signaling all display pronounced daily rhythms, contributing to tissue integrity and metabolic efficiency (11, 12).

Over the past century, technological and societal changes have profoundly altered human exposure to environmental time cues. Artificial lighting, digital technologies, and round-the-clock economic activities have extended human behavior into the biological night and weakened synchronization between endogenous circadian rhythms and environmental cycles (13). As a result, circadian disruption has become increasingly prevalent. A key manifestation is circadian misalignment, defined as a mismatch between endogenous circadian timing and external behavioral or environmental cycles (14). This may occur in several contexts, including shift work, irregular sleep, and inconsistent meal timing. A related phenomenon is social jet lag, referring to discrepancies between sleep timing on workdays and free days (15). Throughout this review, these terms are used as defined here and are not used interchangeably with sleep restriction (insufficient sleep duration) or irregular eating (variable meal timing), though these phenomena frequently co-occur in real-world settings.

Accumulating evidence indicates that chronic circadian misalignment is associated with a wide range of adverse health outcomes, including metabolic syndrome, type 2 diabetes, cardiovascular disease, and immune dysfunction (16, 17). Emerging research further suggests that the musculoskeletal system is also sensitive to circadian disruption. Experimental studies indicate that circadian misalignment can alter bone turnover dynamics, impair skeletal muscle metabolism, and exacerbate inflammatory responses in connective tissues (18, 19). Observational studies further suggest that shift workers may exhibit increased risks of osteoporosis, reduced muscle strength, and greater fracture susceptibility (11, 20, 21). However, in populations such as shift workers, circadian misalignment co-occurs with sleep restriction, reduced exercise, altered diet quality, and occupational biomechanical strain, making it challenging to isolate circadian-specific effects.

In parallel, growing attention has been directed toward the temporal aspects of nutrition. Dietary intake functions not only as a source of metabolic substrates but also as an important zeitgeber capable of entraining peripheral circadian clocks (22). The field of chrononutrition investigates how the timing, frequency, and composition of food intake interact with circadian biology to influence metabolic regulation and health outcomes (23). Most existing reviews have focused on metabolic, cardiovascular, or sleep outcomes. Reviews specifically addressing circadian misalignment in the context of musculoskeletal physiology, or integrating chrononutrition with bone, muscle, and cartilage biology, remain limited. The present narrative review aims to fill this gap by integrating current evidence across circadian biology, dietary behavior, and musculoskeletal physiology. A particular emphasis is placed on distinguishing mechanistic evidence from animal and cellular models from human physiological studies and clinical outcome data, and on explicitly acknowledging where evidence remains preliminary or extrapolated.

## 2 Methods

This manuscript is a narrative review. A systematic literature search was conducted in PubMed/MEDLINE, Scopus, and Web of Science using the following search terms in combination: “circadian rhythm,” “circadian disruption,” “circadian misalignment,” “social jet lag,” “shift work,” “time-restricted eating,” “chrononutrition,” “bone remodeling,” “osteoporosis,” “skeletal muscle,” “sarcopenia,” “cartilage,” “osteoarthritis,” “tendon,” “gut microbiota,” and “musculoskeletal.” Searches were conducted without language restrictions and covered publications up to April 2026. Reference lists of identified articles were also manually screened. Inclusion criteria were: (1) studies reporting on circadian biology, meal timing, or sleep disruption in relation to musculoskeletal tissues or outcomes; (2) original research articles (randomized controlled trials [RCTs], prospective or cross-sectional cohort studies, experimental animal or cell studies) or high-quality systematic reviews and meta-analyses; and (3) full-text availability. Studies were excluded if they addressed circadian biology solely in the context of non-musculoskeletal disease without relevant mechanistic or translational relevance. Evidence was narratively synthesized and, where possible, graded by study design. Throughout this review, we explicitly distinguish findings from RCTs, controlled laboratory studies in humans, prospective cohort studies, cross-sectional studies, and animal or cellular models, as the certainty of evidence varies substantially across these designs.

## 3 Circadian misalignment in modern lifestyles

**Figure 1:**
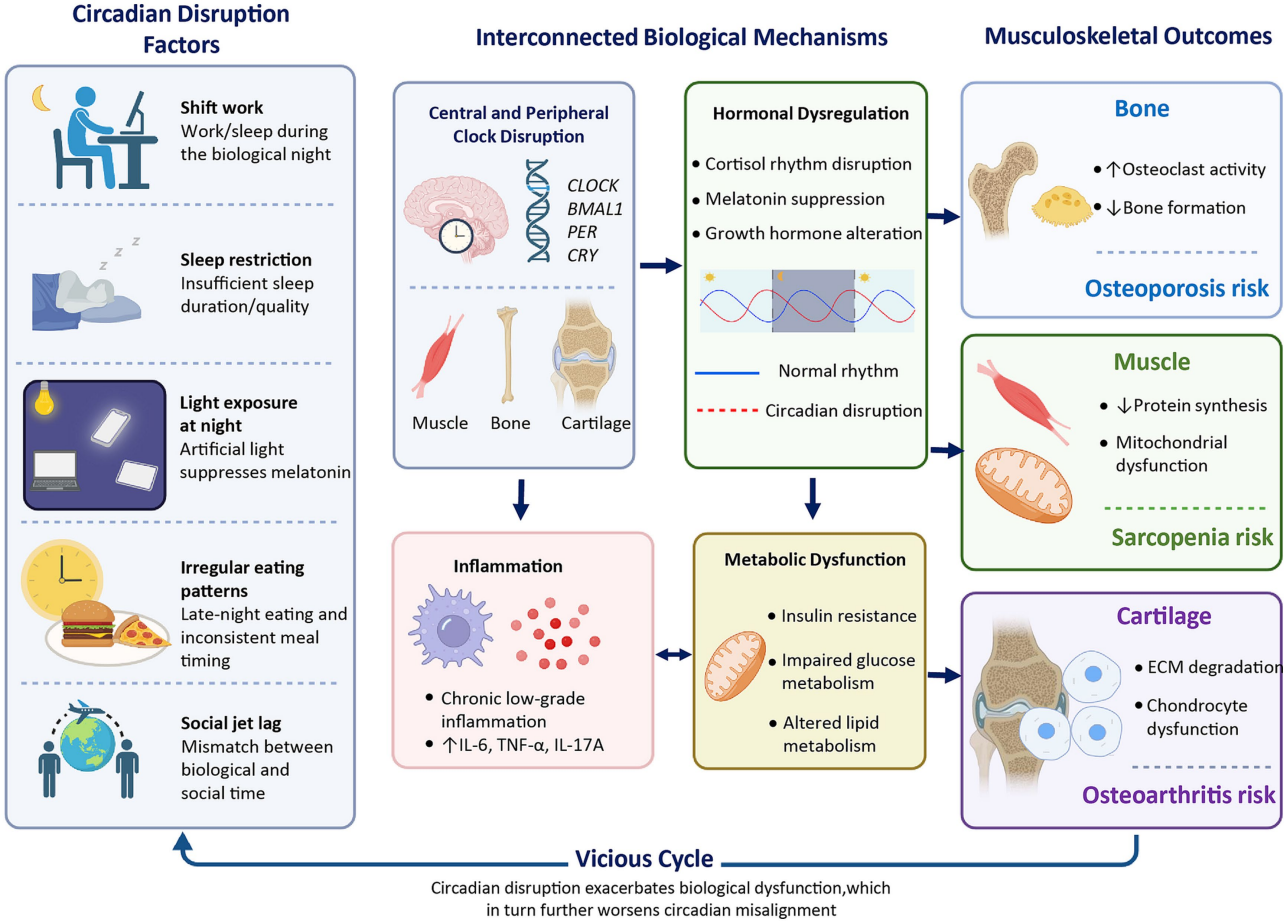
Circadian disruption factors, interconnected biological mechanisms, and musculoskeletal outcomes. The left panel illustrates key sources of circadian misalignment in modern lifestyles, including shift work, sleep restriction, light exposure at night, irregular eating patterns, and social jet lag. The central panel depicts the interconnected biological mechanisms through which these factors impair musculoskeletal homeostasis, encompassing central and peripheral clock disruption, hormonal dysregulation, metabolic dysfunction, and chronic low-grade inflammation. The right panel summarizes downstream consequences for bone, skeletal muscle, and cartilage. A vicious cycle at the bottom indicates that the resulting biological dysfunction further worsens circadian misalignment. Created with BioRender.com.

The following sections describe the major sources of circadian misalignment in contemporary society. While these factors are presented separately for clarity, they frequently co-occur and are deeply confounded with one another. The causal attribution of musculoskeletal harm specifically to circadian misalignment—as distinct from sleep restriction, physical inactivity, or poor diet quality—remains a key methodological challenge (Figure 1).

### 3.1 Disruption of light as the primary zeitgeber

**Figure 2:**
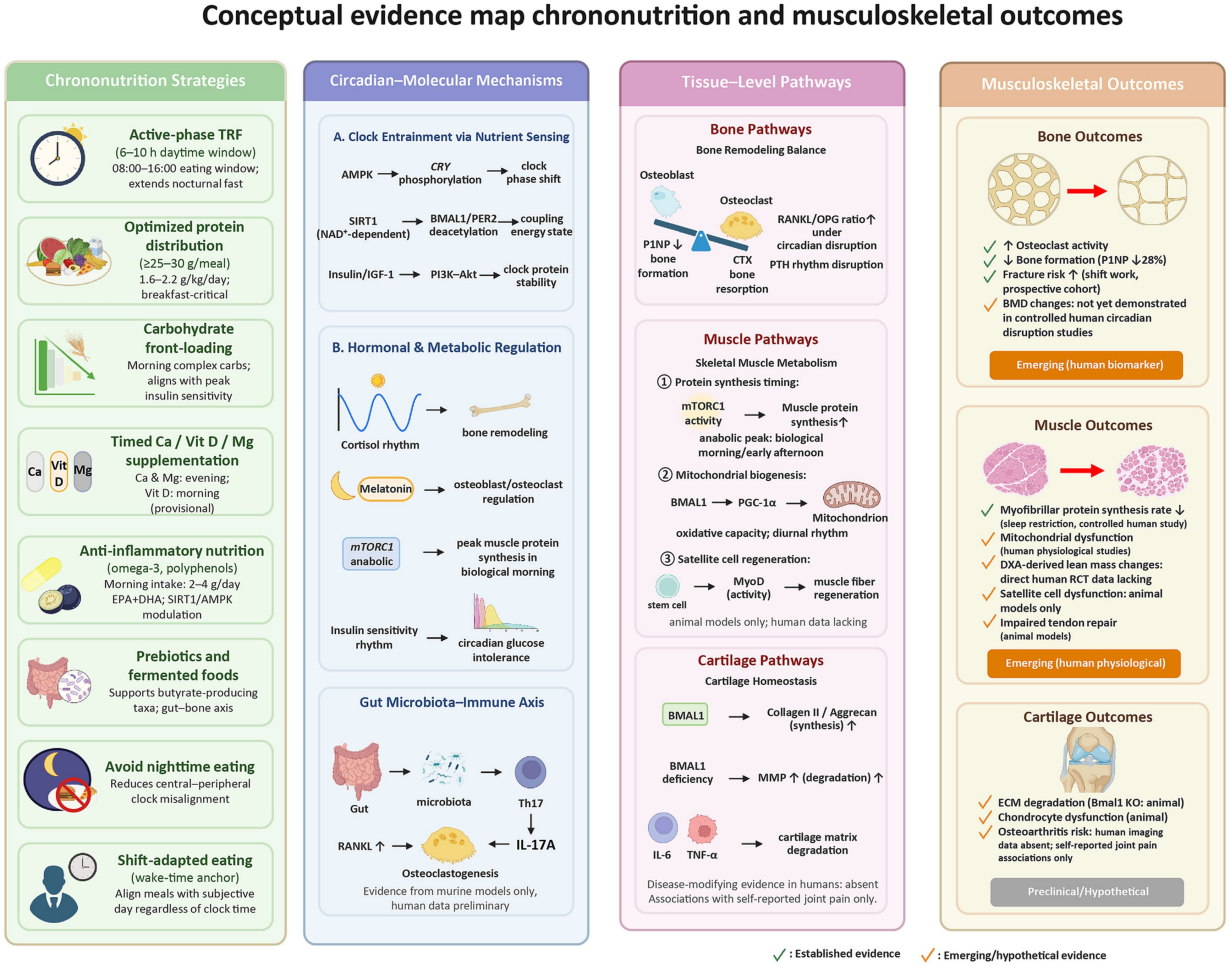
Conceptual evidence map linking chrononutrition strategies to musculoskeletal outcomes via circadian-dependent mechanistic pathways, stratified by evidence level. The figure is organized into four columns: chrononutrition strategies, circadian–molecular mechanisms, tissue-level pathways, and musculoskeletal outcomes. Evidence is stratified using a two-color checkmark system: a green checkmark denotes established evidence supported by controlled human studies, and a yellow checkmark denotes emerging or hypothetical evidence based on short-term human physiological studies, observational cohort data, or animal and mechanistic findings. Created with BioRender.com.

Light represents the most potent zeitgeber for the central circadian pacemaker. Intrinsically photosensitive retinal ganglion cells containing melanopsin project directly to the SCN via the retinohypothalamic tract, transmitting information about environmental light intensity independently of conscious vision (24–26). The relevance of light exposure to musculoskeletal health is indirect but important: by perturbing the central pacemaker, altered light environments drive downstream dysregulation of endocrine, metabolic, and immune pathways that affect bone, muscle, and connective tissue (Figure 2).

#### 3.1.1 Artificial light exposure and melatonin suppression

Evening and nighttime exposure to artificial light, particularly short-wavelength emissions from light-emitting diodes and electronic screens, potently suppresses pineal melatonin secretion (27). Melatonin, with peak production during the biological night, functions as a critical circadian timing signal and is expressed on osteoblasts and osteoclasts, directly stimulating osteoblast differentiation while suppressing osteoclast activation (28–30). A controlled study reported that exposure to 30 lux resulted in more than 50% melatonin suppression at the group level, with individual variability reaching beyond 50% even under dim reading light (10 lux) (30). An international cross-sectional study found that more than 95% of young people use electronic devices within an hour before bedtime (31). Chronic melatonin suppression also impairs antioxidant defenses and promotes pro-inflammatory cytokine production (32, 33), potentially contributing to—though not yet causally established as the direct driver of—circadian-related bone loss independent of sleep effects (34, 35).

#### 3.1.2 Insufficient daylight exposure

Modern indoor environments typically provide 100–500 lux, compared with 1,000–10,000 lux outdoors, even on overcast days (36, 37). Adequate daytime light is critical for advancing circadian phase, reinforcing circadian amplitude, and optimizing resilience to evening light disruption (38). Chronic dim light conditions lead to attenuated circadian amplitude, creating rhythms more susceptible to perturbation by non-photic zeitgebers such as irregular meals, social activities, or shift work schedules (39, 40).

### 3.2 Social and occupational timing

#### 3.2.1 Shift work

Shift work, defined as work schedules outside approximately 07:00–18:00, affects approximately 15–25% of the workforce in industrialized nations (41). Night shift work requires workers to be active during their biological night and to sleep during their biological day, creating severe circadian strain that rarely resolves even after years of exposure (42). Gene expression rhythms in peripheral tissues may become uncoupled from the central SCN rhythm, which often remains entrained to the light–dark cycle through daytime light exposure, creating persistent internal desynchrony (16, 43). Observational studies have documented associations between shift work and increased osteoporosis risk, fracture incidence, low back pain, and musculoskeletal injuries (11, 12, 20). These associations, however, are confounded by concurrent sleep restriction, physical inactivity, dietary changes, and occupational biomechanics, as discussed in Section 3.4.

#### 3.2.2 Social jet lag

Social jet lag refers to the misalignment between biological time and social time imposed by work or social obligations, quantified as the difference in mid-sleep timing between free days and workdays (44). Up to 70% of the general population experiences at least 1 hour of social jet lag, with younger adults and later chronotypes showing greater misalignment (45). Large epidemiological studies have associated social jet lag with impaired glucose regulation, elevated C-reactive protein, and increased risk of metabolic syndrome (46–49). Direct human studies on social jet lag with validated musculoskeletal endpoints (bone mineral density, lean mass, fracture incidence) are currently limited, though the shared mechanisms with shift work suggest plausible musculoskeletal consequences warranting prospective investigation.

### 3.3 Irregular eating patterns

Meal timing represents the most potent non-photic zeitgeber for peripheral circadian oscillators, particularly in metabolic tissues (22). Modern irregular eating patterns—breakfast skipping, late-night snacking, and highly variable meal times—send conflicting temporal signals to peripheral organs, potentially inducing desynchrony between central and peripheral oscillators (50). Night eating syndrome affects 0.5–1.5% of the general population but up to 25% of obese individuals (51), and the normalization of late-night eating creates widespread peripheral clock disruption with metabolic consequences (52).

### 3.4 Confounding and causal inference

A critical methodological challenge is the separation of circadian-specific effects from the many co-occurring exposures that accompany real-world circadian disruption. In shift workers, circadian misalignment co-occurs with reduced and fragmented sleep, altered dietary intake, reduced physical activity, occupational ergonomic strain, and psychosocial stress. Most cross-sectional and cohort studies cannot fully disentangle these factors even with statistical adjustment, because many are mediators rather than independent confounders. Controlled laboratory studies using forced-desynchrony protocols—which allow circadian and sleep–wake processes to run at different periods while keeping sleep duration and diet constant—provide the strongest evidence for circadian-specific effects on outcomes such as bone turnover markers and insulin resistance (16, 53). Such designs, however, are resource-intensive, short-term, and conducted predominantly in healthy young adults, limiting generalizability. Future research should prioritize designs that can isolate circadian phase from sleep duration, caloric intake, activity level, and diet quality, and should account for chronotype, sex, menopausal status, and age as important effect modifiers.

## 4 Circadian rhythms and musculoskeletal physiology

### 4.1 The molecular clock machinery

The circadian system comprises a hierarchical network of oscillators. At the apex, the SCN coordinates peripheral tissue clocks through neural (autonomic nervous system), hormonal (melatonin, cortisol, growth hormone), and behavioral (activity, feeding, temperature) outputs (9, 54). The molecular basis involves interlocking transcription–translation feedback loops: CLOCK and BMAL1 heterodimerize and activate expression of Period (PER1–3) and Cryptochrome (CRY1–2) genes, which in turn inhibit CLOCK: BMAL1 activity (10). Accessory loops involving REV-ERBα/*β* and RORα/β add stability (55). Post-translational modifications including phosphorylation, ubiquitination, and acetylation add further regulatory complexity (56, 57). Peripheral tissues—including bone, skeletal muscle, and cartilage—possess autonomous circadian oscillators capable of sustaining rhythms ex vivo (58, 59), additionally entrained by local zeitgebers, particularly feeding time and temperature (60). The hierarchical yet decentralized architecture means that peripheral oscillators can become desynchronized from the master clock, creating internal temporal disorganization that may be more detrimental than a uniform phase shift (16, 61).

### 4.2 Crosstalk between muscle and bone circadian clocks

An important dimension of circadian musculoskeletal biology is the bidirectional communication between skeletal muscle and bone, mediated by endocrine and paracrine signals that are themselves under circadian control. Skeletal muscle secretes myokines including irisin (FNDC5) and myostatin in a PGC-1α/BMAL1-dependent manner (62). Irisin stimulates osteoblast differentiation in animal and *in vitro* models, while myostatin, a negative regulator of muscle mass, also inhibits osteoblastogenesis; both exhibit diurnal variation in preclinical studies. Conversely, bone-derived osteocalcin, secreted by osteoblasts under circadian regulation, promotes muscle glucose uptake via GPRC6A receptors on myofibers, potentially optimizing muscle fuel utilization during the active phase (63). Additionally, sclerostin secreted by osteocytes in a mechanically and circadian-regulated manner modulates Wnt signaling in both bone and muscle. Circadian disruption of osteocyte clock function may therefore alter sclerostin secretion patterns, impacting both bone formation and muscle anabolic signaling simultaneously. Furthermore, peak muscle contractile force occurs during the active phase in alignment with circadian physiology, stimulating bone mechanotransduction and remodeling. Circadian disruption that impairs muscle contractile capacity may secondarily reduce mechanical loading of bone, contributing to bone loss through an indirect pathway. This muscle–bone functional unit is likely disrupted in both sarcopenia and osteoporosis, where the two conditions frequently co-occur as “osteosarcopenia,” and circadian misalignment may represent a shared upstream determinant.

### 4.3 Circadian regulation of bone biology

#### 4.3.1 Cellular clocks in skeletal cells

All major bone cell types—osteoblasts, osteoclasts, and osteocytes—express functional molecular clocks (64). BMAL1 knockout mice exhibit decreased bone volume fraction, trabecular thinning, impaired osteoblast differentiation, and decreased bone formation rates (65). BMAL1 regulates osteoblastogenesis through Wnt signaling components (Wnt10b, Lrp5), reactive oxygen species homeostasis, and mitochondrial function (66). Osteoblast-specific BMAL1 deletion recapitulates many features of the global knockout, confirming cell-autonomous clock functions in bone formation (66, 67). Conversely, PER and CRY deficient mice demonstrate increased bone mass associated with enhanced osteoblast proliferation and reduced osteoclastogenesis—findings reflecting the complex, phase-specific functions of the molecular clock (68, 69). The circadian clock in osteoclast precursors also regulates RANKL sensitivity and fusion dynamics (18, 19). Osteocytes express circadian clocks that regulate mechanotransduction and endocrine function, coordinating the activity of surface osteoblasts and osteoclasts (6, 67).

#### 4.3.2 Bone remodeling rhythms and their disruption

Bone remodeling exhibits pronounced circadian rhythmicity (6). Markers of bone resorption (e.g., CTX) peak during the early morning hours, while bone formation markers (e.g., P1NP) peak in the afternoon or evening. RANKL shows circadian oscillation in osteoblasts while its decoy receptor OPG shows counter-phase oscillation, gating osteoclastogenesis across the day (6, 19). Parathyroid hormone (PTH) and cortisol rhythms further contribute to remodeling patterns (70). Shift workers demonstrate flattened diurnal variation in bone turnover markers, with impaired coupling between formation and resorption (71). In a controlled forced-desynchrony study (the strongest human evidence design), 3 weeks of circadian misalignment combined with sleep restriction reduced P1NP by approximately 28% in young men without significant changes in CTX, indicating uncoupling of bone turnover (53). This suppression persisted throughout the intervention period (72). It should be noted that validated clinical endpoints (bone mineral density, fracture incidence) have not yet been examined under controlled circadian misalignment in humans; the clinical significance of these biomarker changes therefore requires further investigation.

#### 4.3.3 The gut microbiota–bone axis

Emerging evidence from animal models implicates the gut microbiota as a mediator of circadian–musculoskeletal interactions (73). The intestinal microbiome exhibits robust circadian rhythmicity influenced by both host circadian clocks and feeding patterns (74, 75). In murine models, rest-phase time-restricted feeding induces dysbiosis characterized by expansion of pro-inflammatory taxa, promoting intestinal Th17 cell accumulation and systemic IL-17A elevation, which facilitates osteoclastogenesis through RANKL-dependent mechanisms (76–78). Antibiotic depletion of microbiota, genetic ablation of IL-17A signaling, or fecal microbiota transplantation from misaligned donors confirms that microbiota composition mediates skeletal effects in these animal models (78–80). Direct evidence for this gut microbiota–Th17–osteoclast axis in human circadian disruption remains preliminary; these findings nonetheless establish an important conceptual therapeutic target and motivate future investigation of microbiota-focused nutritional interventions in humans.

### 4.4 Circadian regulation of muscle physiology

#### 4.4.1 Muscle protein synthesis and degradation

Skeletal muscle shows circadian variation in protein metabolism that contributes to long-term muscle mass maintenance (81, 82). Evidence from animal models, particularly zebrafish, suggests muscle growth can exhibit circadian regulation independent of locomotor activity (83). In adult mammals, direct evidence for robust circadian oscillation of muscle protein synthesis independent of feeding or activity is more limited; available data suggest that anabolic responsiveness to amino acids, insulin, and resistance exercise tends to peak during the biological morning/early afternoon (83, 84). This distinction is important: much of the apparent circadian variation in muscle anabolism may reflect circadian modulation of feeding and activity rather than an autonomous muscle clock effect on protein synthesis per se. BMAL1 influences translational machinery and amino acid transport in some contexts, while PER proteins can inhibit mTORC1 via TSC1 recruitment, supporting rhythmic anabolic control (85, 86). Muscle-specific BMAL1 disruption leads to metabolic defects and blunted responses to anabolic stimuli without causing overt atrophy under basal conditions (87), while global Bmal1 knockout produces profound atrophy largely reflecting developmental or extra-muscular effects (88, 89). Proteolytic pathways show higher activity during rest and fasting, aiding quality control and amino acid recycling (90, 91). Circadian misalignment may disrupt this coordination (92), though human studies providing direct evidence of circadian disruption on clinically meaningful muscle outcomes such as Dual-energy X-ray Absorptiometry (DXA)-derived lean mass or functional strength remain limited (93).

#### 4.4.2 Metabolic flexibility and mitochondrial function

Muscle metabolic flexibility—the capacity to switch between lipid and glucose oxidation—is fundamentally circadian-regulated (94). BMAL1 regulates mitochondrial biogenesis through coactivation of PGC-1α, the master regulator of mitochondrial gene expression (62). Mitochondrial respiratory capacity peaks during the active phase, aligned with physical activity and feeding (95). Circadian misalignment impairs metabolic flexibility through reduced BMAL1 expression, altered timing of substrate availability, and insulin resistance (94, 96). Lipid accumulation in muscle produces lipotoxicity and promotes inflammation (97), while reduced ATP availability impairs contractile function (98). These mechanisms are supported primarily by animal models and short-term human physiological studies; the contribution of circadian-specific metabolic impairment to clinically diagnosed sarcopenia in prospective human cohorts has not been directly established.

#### 4.4.3 Satellite cells and regenerative capacity

Satellite cells—muscle stem cells that activate in response to injury—exhibit circadian variation in proliferative and differentiation capacity during the active phase (99). The transition from quiescence to activation is gated by circadian regulation of metabolic enzymes (100, 101), and myogenic regulatory factors such as MyoD show circadian variation in differentiation efficiency (102). Disruption of satellite cell clocks impairs muscle regeneration following injury and promotes fibrotic deposition (99, 103). These findings derive primarily from animal models; direct evidence that circadian misalignment impairs muscle regeneration in humans remains lacking, though this represents a clinically important hypothesis.

### 4.5 Joint and connective tissue rhythms

#### 4.5.1 Articular cartilage and inflammatory rhythms

Chondrocytes express functional molecular clocks, with BMAL1 regulating expression of matrix components (collagen II, aggrecan) and degradative enzymes (matrix metalloproteinases), with the balance between anabolic and catabolic processes varying across the day (63). Inflammatory joint diseases demonstrate prominent circadian symptomatology: rheumatoid arthritis characteristically shows morning stiffness and pain, reflecting nocturnal peaks in inflammatory cytokines (IL-6, TNF-*α*) driven by circadian regulation of immune cell trafficking (104, 105). Circadian misalignment may amplify inflammatory responses, potentially exacerbating disease activity in inflammatory arthritis (106). Animal models (chondrocyte-specific Bmal1 knockout) demonstrate that clock gene disruption leads to cartilage degeneration and accelerated osteoarthritis-like pathology (107, 108). In humans, evidence linking circadian disruption to incident osteoarthritis or cartilage loss on imaging is currently absent; available data are restricted to associations between shift work or sleep disturbance and self-reported joint pain, which do not establish disease modification.

#### 4.5.2 Tendon and ligament biology

Tendons also exhibit circadian biology: collagen synthesis shows circadian variation with peak production during the active phase, and clock genes regulate fibroblast proliferation and collagen secretion (109, 110). Animal studies suggest that healing following tendon injury may differ based on injury timing relative to circadian phase (111). The avascular nature of tendons and scarcity of direct human evidence limit clinical translation; recommendations regarding the timing of tendon-directed nutritional interventions should be regarded as hypotheses requiring empirical validation (Table 1).

**Table 1:** Summary of evidence linking circadian disruption to musculoskeletal disorders.

Category	Study type	Model/population	Circadian factor	Key findings	Outcome	References
Bone	Animal (mouse)	Bmal1 knockout mice	Clock gene disruption	Premature aging syndrome: sarcopenia, cataracts, organ shrinkage	Premature aging phenotype	(172)
Bone	Animal (mouse)	Bmal1 knockout mice	Clock gene disruption	Impaired osteoblast differentiation	Bone loss	(66)
Bone	Prospective cohort (human)	Nurses’ Health Study	Shift work (night)	Increased hip fracture risk	Fracture risk	(20)
Muscle	Animal (mouse)	Bmal1 knockout mice	Clock gene disruption	Muscle weakness, mitochondrial dysfunction	Sarcopenia-like phenotype	(173)
Muscle	Controlled lab study (human)	Healthy young men; sleep restriction	Sleep restriction	Reduced myofibrillar protein synthesis rate	Muscle loss risk	(174)
Cartilage	Animal (mouse)	Chondrocyte Bmal1 knockout	Clock gene disruption	Cartilage degeneration, OA-like pathology	Osteoarthritis progression	(107)
Cartilage	Animal (mouse)	Murine OA model	Clock gene rhythms	Autonomous circadian clock regulates cartilage homeostasis genes	Osteoarthritis progression	(108)

## 5 Diet–circadian interactions

### 5.1 Feeding as a zeitgeber: mechanisms and phase dependence

While light dominates central SCN entrainment, feeding time serves as the principal zeitgeber for peripheral circadian oscillators in liver, gastrointestinal tract, pancreas, adipose tissue, and muscle (112, 113). Key nutrient-sensing pathways include: AMP-Activated Protein Kinase (AMPK), which responds to low cellular energy status by phosphorylating CRY and shifting clock phase (114); Sirtuin 1 (SIRT1), an NAD + -dependent deacetylase that deacetylates BMAL1 and PER2, coupling cellular energy status to circadian function (115, 116); and Insulin/IGF-1 Signaling, which activates PI3K–Akt pathways that modulate clock protein stability (117). Food intake during the biological day produces robust phase shifts, while feeding during the biological night may produce inappropriate phase shifts and incomplete peripheral clock entrainment (118).

### 5.2 Chrononutrition: temporal patterns and metabolic consequences

#### 5.2.1 Breakfast protein consumption

Morning protein intake (≥30 g providing ≥2–3 g leucine) has been associated with enhanced muscle protein synthesis in some studies, mediated by circadian modulation of mTORC1 signaling primed for anabolic responses during the biological morning (119, 120). A randomized controlled trial in older adults demonstrated that protein distribution skewed toward breakfast enhanced whole-body protein retention and muscle mass accretion compared with evening-dominant distribution (121). However, studies directly comparing morning versus evening protein in healthy younger adults with DXA-confirmed lean mass endpoints are more limited, and the magnitude of the timing advantage may be smaller in non-sarcopenic individuals. Breakfast skipping eliminates this priming effect and may impair all-day protein balance (122).

#### 5.2.2 Evening carbohydrate effects

Late-day carbohydrate consumption impairs glucose tolerance and promotes hepatic lipid accumulation through circadian modulation of insulin sensitivity and glucose uptake (123, 124). The same carbohydrate load produces greater glycemic excursion when consumed at night compared to morning—“circadian glucose intolerance (125).” Nighttime carbohydrate consumption may favor hepatic fat accumulation, producing systemic insulin resistance and inflammatory cytokine release that secondarily influence muscle insulin sensitivity (126, 127). These metabolic effects are supported by controlled human physiological studies but have not yet been directly linked to changes in bone mineral density or clinically diagnosed sarcopenia.

#### 5.2.3 Meal frequency and fasting windows

Consolidated intake into discrete feeding windows may reinforce circadian rhythms by creating clear transitions between anabolic (fed) and catabolic (fasted) states (128). However, prolonged fasting without adequate protein may compromise muscle protein balance, particularly in older adults with blunted anabolic responses (129). The optimal balance between fasting duration and feeding frequency likely varies by age, health status, and activity level, and should not be extrapolated uniformly across populations.

### 5.3 Circadian variation in nutrient utilization

#### 5.3.1 Protein and amino acids

The circadian peak in anabolic hormone secretion and mTORC1 sensitivity may modulate the anabolic response to protein intake, suggesting that protein timing relative to activity could matter for optimizing muscle adaptation (130, 131). However, human interventional data directly demonstrating that morning versus evening protein intake produces superior DXA-confirmed lean mass gains in controlled trials remain limited; most evidence derives from observational, short-term physiological, or animal studies.

#### 5.3.2 Calcium and vitamin D

Calcium absorption shows circadian variation, with active transport mechanisms peaking during the day (132). The vitamin D receptor exhibits circadian expression in certain tissues, and preclinical evidence suggests circadian regulation of intestinal calcium handling (132, 133). The hypothesis that supplementation timing influences bone-relevant outcomes requires prospective clinical testing; current evidence is insufficient to make firm timing recommendations.

#### 5.3.3 Omega-3 fatty acids and polyphenols

Anti-inflammatory effects of these bioactive compounds may be phase-dependent, given the circadian variation in inflammatory cytokine expression (134–136). Direct comparative human trials examining morning versus evening supplementation with musculoskeletal endpoints are lacking; timing recommendations therefore remain hypothetical.

## 6 Nutritional adaptation strategies

Based on mechanistic understanding of diet–circadian interactions, practical nutritional strategies can be designed to adapt to or mitigate circadian misalignment (137). The evidence base for specific musculoskeletal benefit varies substantially across interventions. Many recommendations derive primarily from metabolic, cardiovascular, or general health studies rather than trials with validated musculoskeletal endpoints. Where human evidence is limited or restricted to specific populations, this is explicitly stated.

### 6.1 Time-restricted feeding: principles and musculoskeletal effects

Time-restricted feeding (TRF), limiting daily food intake to a consistent window typically of 6–10 h, has emerged as a promising strategy for circadian realignment and metabolic health (138). By consolidating feeding signals and extending the nocturnal fast, TRF reinforces circadian coherence, enhances autophagy, and improves metabolic flexibility (78).

#### 6.1.1 Active-phase TRF: protective effects

In murine models, TRF aligned with the biological active phase prevents bone loss observed with rest-phase feeding, potentially by reinforcing circadian rhythms, reducing inflammatory tone, and enhancing autophagic clearance (78). Human studies of early TRF (eating window approximately 08:00–16:00) demonstrate preservation or improvement in muscle strength and body composition, with neutral or positive effects on lean mass when protein intake is adequate (139, 140). These trials have generally been conducted in healthy adults over weeks to months and have used body composition rather than validated clinical musculoskeletal endpoints; generalizability to frail older adults, individuals with chronic disease, or shift workers requires further study.

#### 6.1.2 Rest-phase TRF: risks and considerations

Conversely, rest-phase TRF—eating during the biological night—may exacerbate circadian-related pathology. In murine models, rest-phase TRF induces significant bone loss through the gut microbiota–Th17–osteoclast axis; notably, female mice showed no such phenotype in the same studies (78). This sex-specific vulnerability in the murine model likely reflects interactions between estrogen signaling and circadian clock function. Translating this finding to humans requires caution; postmenopausal women may constitute a higher-risk group, but direct human evidence linking rest-phase eating specifically to bone loss has not been established (21).

#### 6.1.3 Practical implementation guidelines

For diurnally active individuals, early TRF (approximately 07:00–15:00 or 08:00–16:00) may optimize alignment with circadian physiology (141). Important caveats apply: early TRF may be inappropriate for frail older adults who struggle to meet protein targets within a narrow window, underweight individuals, those with eating disorder history, and pregnant or lactating individuals. For night shift workers, potential strategies include: (i) maintaining a consistent eating window relative to wake time regardless of clock time (142); (ii) minimizing intake during the first half of the night shift (118); (iii) consuming the main meal at the beginning of the subjective day (143); and (iv) fasting during the subjective night sleep period (144). These strategies are supported primarily by short-term metabolic studies; their effect on musculoskeletal outcomes in shift workers has not been directly evaluated.

### 6.2 Macronutrient timing strategies

#### 6.2.1 Protein distribution and quality

Optimizing protein distribution across meals represents a practical adaptation: providing ≥25–30 g per meal (1.6–2.2 g/kg/day total) distributed to exploit circadian peaks in anabolic sensitivity (121). For individuals with conventional schedules, even distribution across breakfast, lunch, and dinner maximizes 24-h muscle protein synthesis, with breakfast protein particularly critical after the overnight catabolic fast (145). For shift workers, maintaining protein distribution relative to wake time may help preserve muscle circadian function; absolute protein quantity may need to be modestly increased to compensate for reduced circadian sensitivity (118). Leucine-rich sources most effectively stimulate mTORC1; plant proteins may require higher intake to achieve equivalent leucine thresholds (146). These recommendations are supported by RCTs in older adults but largely extrapolated for other populations.

#### 6.2.2 Carbohydrate periodization

Morning complex carbohydrate consumption, coinciding with peak insulin sensitivity and physical activity, supports muscle glycogen replenishment and anabolic signaling (147). Reducing evening carbohydrate intake may mitigate circadian-related metabolic dysfunction (148). High-glycemic foods produce greater metabolic disruption when consumed at night, suggesting lower-glycemic sources are preferable if nighttime carbohydrates are necessary (149). Recommendations about carbohydrate periodization for musculoskeletal outcomes specifically are extrapolated from metabolic studies; prospective trials with bone or muscle endpoints are needed.

### 6.3 Micronutrient chronobiology and bioactive compounds

#### 6.3.1 Vitamin D

Vitamin D deficiency—common in shift workers with limited daylight exposure—is associated with sleep disorders and aberrant clock gene expression (150, 151). Morning supplementation may theoretically align with endogenous production rhythms. However, human trials comparing morning versus evening vitamin D supplementation with bone mineral density or fracture as the primary endpoint are lacking; current timing recommendations are therefore hypothetical.

#### 6.3.2 Calcium

The theoretical rationale for evening calcium supplementation—to align with nocturnal bone resorption peaks—has some support from small trials (152, 153). Calcium carbonate requires acid for absorption and may be less effective in the evening when gastric acid secretion is reduced; calcium citrate may be preferable for nighttime dosing. Confirmatory trials with bone mineral density endpoints are needed before strong timing recommendations can be made.

#### 6.3.3 Magnesium

Evening supplementation (200–400 mg magnesium glycinate or citrate) may enhance sleep architecture and provide indirect musculoskeletal benefits through improved rest and hormone secretion during sleep (154). Evidence specific to musculoskeletal outcomes in circadian-disrupted individuals remains limited.

#### 6.3.4 Omega-3 fatty acids (EPA/DHA)

Anti-inflammatory effects may align with morning inflammatory peaks; doses of 2–4 g/day EPA + DHA may be necessary for anti-inflammatory effects (155, 156). The recommendation for morning omega-3 supplementation should be regarded as a hypothesis pending confirmatory trials.

#### 6.3.5 Polyphenols

Found in colorful fruits, vegetables, green tea, coffee, and spices, polyphenols modulate clock gene expression via SIRT1 and AMPK activation and exhibit antioxidant and anti-inflammatory properties (157, 158). Curcumin, resveratrol, and quercetin have shown clock-modulating effects in preclinical studies; human bioavailability remains a challenge, and direct evidence for musculoskeletal benefit from timed polyphenol supplementation is currently lacking.

#### 6.3.6 Prebiotics and fermented foods

Fermentable fibers and fermented foods (yogurt, kefir, sauerkraut, kimchi) may support butyrate-producing taxa and intestinal barrier integrity, potentially modulating the gut microbiota–bone axis established in animal models (76, 159). Evidence specific to circadian-related dysbiosis and musculoskeletal outcomes in humans remains preliminary; these dietary practices are recommended as part of a generally healthful diet pending definitive clinical trials.

### 6.4 Targeted interventions for specific misalignment patterns

#### 6.4.1 Permanent night shift workers

Most interventions focus on reducing circadian misalignment rather than achieving full circadian inversion (160). Evidence-graded strategies include: Light Management (controlled physiological studies): bright light during the night shift promotes alertness and delays circadian phase (161, 162); Melatonin Supplementation (meta-analyses of sleep outcomes): taken prior to planned daytime sleep may facilitate sleep onset (163); Meal Timing (short-term metabolic studies): aligning the main meal with the waking period may stabilize metabolic rhythms, and lighter meals during night shifts are better tolerated (160, 164); Caffeine (alertness studies): strategic use during the early portion of the night shift can maintain alertness (161); Exercise (general health studies): moderate activity during the waking period promotes wellbeing, while vigorous activity before sleep may interfere with sleep initiation (165). None of these strategies has been demonstrated to reduce musculoskeletal disease burden (fracture, incident sarcopenia) in shift workers in controlled trials.

#### 6.4.2 Rotating shift workers

The use of an anchor sleep period—maintaining a portion of daily sleep at a consistent time—may help preserve some degree of circadian regularity (166). Some studies suggest rapid rotation may reduce prolonged misalignment, though findings remain inconsistent (167). No interventional data on musculoskeletal outcomes in rotating shift workers are currently available.

#### 6.4.3 Social jet lag mitigation

Strategies focus on maintaining consistent behavioral rhythms: stable sleep and wake times across workdays and weekends, consistent daily meal schedules, and regular morning light exposure (168–170). These behavioral strategies are supported by observational studies linking sleep regularity to metabolic health; prospective evidence for musculoskeletal benefit has not been established.

#### 6.4.4 Age-, sex-, and chronotype-specific considerations

The evidence base is disproportionately derived from studies in young, healthy, predominantly male adults. Older adults have blunted anabolic responses to protein, reduced circadian amplitude, and greater susceptibility to sarcopenia and osteoporosis, making circadian-aligned protein distribution particularly important while simultaneously raising concerns about the feasibility of narrow TRF windows that may impair adequate intake (129). Women, particularly postmenopausal, face heightened skeletal vulnerability due to estrogen deficiency; murine evidence suggests sex-specific differences in the skeletal response to rest-phase feeding, and estrogen–clock interactions may render postmenopausal women at greater risk from circadian disruption (21). Individuals with late chronotypes face inherent social jet lag even without shift work; rigid early TRF may be poorly aligned with their internal circadian time. Future clinical trials should stratify by sex, menopausal status, chronotype, and age, and report evidence separately for these subgroups (Table 2).

**Table 2:** Chrononutrition strategies for mitigating circadian disruption–induced musculoskeletal impairment.

Intervention	Core principle	Mechanism	Evidence type	Human musculoskeletal endpoint	Limitations/cautions	References
Active-phase TRF (6–10 h daytime window)	Consolidate intake to biological day	Synchronizes peripheral clocks, enhances autophagy	Animal models; short-term human RCTs (body composition)	Body composition (lean mass); no fracture/ bone mineral density RCTs	Caution in frail elderly, underweight, eating disorder history, pregnancy; protein adequacy must be confirmed	(137, 139, 141)
Avoid nighttime eating	Minimize intake during biological night	Reduces central–peripheral clock misalignment	Controlled human physiological studies; animal models	Glucose tolerance, BMI; no direct bone/muscle endpoint RCTs	Compliance difficult in shift workers; nighttime eating unavoidable in some contexts	(141, 143)
Shift-adapted eating (wake-time anchor)	Align meals with subjective day regardless of clock time	Re-establishes metabolic rhythmicity relative to behavioral phase	Short-term controlled studies (metabolic outcomes)	Glucose tolerance, gastrointestinal symptoms; no bone/muscle endpoint trials	Practically challenging; needs prospective evaluation with musculoskeletal endpoints	(131, 143)
Optimized protein distribution (≥25–30 g/meal; 1.6–2.2 g/kg/day)	Protein at each meal including breakfast	Activation of mTORC1 at circadian anabolic peaks	RCTs in older adults (lean mass); physiological studies	Lean mass (DXA) in older adults; protein synthesis rate	Evidence strongest in older adults; plant proteins may require higher quantities	(121, 129)
Carbohydrate front-loading	Higher carb intake earlier in day	Matches circadian peak in insulin sensitivity	Controlled human physiological studies; epidemiological associations	Glucose tolerance, metabolic biomarkers; no bone/muscle endpoint trials	Extrapolated from metabolic studies; evening exercise training requires peri-exercise carbohydrate	(147, 148)
Timed Ca/Vit D/Mg supplementation	Align with circadian absorption and secretion rhythms	Ca: limits nocturnal resorption; Vit D: supports clock–bone axis; Mg: supports sleep and melatonin synthesis	Small human trials (bone resorption markers); preclinical; observational	Bone resorption markers (Ca); no bone mineral density /fracture RCTs for timing	Timing recommendations are provisional hypotheses; Ca carbonate less effective at night (prefer citrate); confirmatory trials needed	(132, 133, 154)
Anti-inflammatory nutrition (omega-3, polyphenols)	Morning intake to align with peak inflammatory tone	Suppresses inflammatory signaling; SIRT1/AMPK-mediated clock modulation	Animal/preclinical; limited human observational data	Inflammatory markers only; no musculoskeletal endpoint trials for timed supplementation	Timing recommendations hypothetical; ≥2–4 g/day EPA + DHA for anti-inflammatory effect; polyphenol bioavailability variable	(134, 157)
Prebiotics and fermented foods	Support beneficial gut microbiota	Suppresses Th17–IL-17 axis; maintains intestinal barrier; enhances butyrate production	Animal models; preliminary human observational data	Not yet studied with bone/muscle clinical endpoints in humans	Mechanistic pathway established in rodents only; generally safe as part of a healthful diet	(75, 78)

## 7 Implications for musculoskeletal health and future perspectives

The evidence synthesized in this review highlights circadian misalignment as an important and potentially modifiable determinant of musculoskeletal health. Disruption of circadian rhythms is associated with—and in animal models causally linked to—alterations in bone remodeling, muscle metabolism, and connective tissue homeostasis. In humans, the evidence is strongest for associations between shift work and fracture risk and between sleep restriction and acute reductions in bone formation markers; direct evidence linking circadian misalignment per se to changes in bone mineral density, DXA-derived lean mass, or incident sarcopenia in longitudinal human studies remains limited. Chrononutrition emerges as a key interface through which behavioral factors can either exacerbate or mitigate these disturbances (84).

From a clinical and public health perspective, incorporating temporal dimensions into lifestyle interventions may enhance prevention and management of musculoskeletal disorders. Strategies such as aligning food intake with the biological active phase, optimizing protein distribution, and avoiding late-night eating represent practical, low-cost approaches to support circadian integrity and tissue function (131). These interventions are best regarded as potentially beneficial lifestyle practices with a plausible mechanistic rationale, rather than established treatments. They may be particularly relevant for shift workers, individuals with irregular sleep patterns, and aging populations susceptible to sarcopenia and osteoporosis, though evidence in these specific groups is still emerging (171).

Future research priorities include: (1) RCTs examining chrononutrition interventions with validated clinical musculoskeletal endpoints (bone mineral density by DXA, incident fracture, lean mass, grip strength, incident sarcopenia, and cartilage loss on imaging); (2) controlled laboratory paradigms to isolate circadian-specific effects on musculoskeletal biomarkers from those of sleep restriction, caloric intake, and physical inactivity; (3) adequate representation of women (including pre- and postmenopausal), older adults, and individuals with late chronotypes in intervention trials, with subgroup analyses planned *a priori*; (4) investigation of the gut microbiota–circadian–bone axis in human circadian disruption models; (5) multi-omics approaches to identify circadian biomarkers of musculoskeletal risk; and (6) pragmatic trials in occupational settings to evaluate the feasibility and efficacy of circadian-aligned dietary interventions on musculoskeletal outcomes over years rather than weeks.

In conclusion, integrating circadian biology into musculoskeletal research offers a promising framework for advancing precision prevention and treatment. Chrononutrition and circadian-aligned lifestyle strategies may represent feasible, low-cost interventions with significant potential to reduce the global burden of musculoskeletal disorders. Translating this promise into clinical practice will require rigorous human trials with appropriate musculoskeletal endpoints, attention to individual variability, and careful acknowledgment of the current boundaries of the evidence base.
